# Mediating Effect of Tobacco Dependence on the Association Between Maternal Smoking During Pregnancy and Chronic Obstructive Pulmonary Disease: Case-Control Study

**DOI:** 10.2196/53170

**Published:** 2024-02-22

**Authors:** Jinxuan Li, Jianying Xu, Lan Yang, Yongjian Xu, Xiangyan Zhang, Chunxue Bai, Jian Kang, Pixin Ran, Huahao Shen, Fuqiang Wen, Kewu Huang, Wanzhen Yao, Tieying Sun, Guangliang Shan, Ting Yang, Yingxiang Lin, Jianguo Zhu, Ruiying Wang, Zhihong Shi, Jianping Zhao, Xianwei Ye, Yuanlin Song, Qiuyue Wang, Gang Hou, Yumin Zhou, Wen Li, Liren Ding, Hao Wang, Yahong Chen, Yanfei Guo, Fei Xiao, Yong Lu, Xiaoxia Peng, Biao Zhang, Zuomin Wang, Hong Zhang, Xiaoning Bu, Xiaolei Zhang, Li An, Shu Zhang, Zhixin Cao, Qingyuan Zhan, Yuanhua Yang, Lirong Liang, Bin Cao, Huaping Dai, Kian Fan Chung, Zhengming Chen, Jiang He, Sinan Wu, Dan Xiao, Chen Wang, China Pulmonary Health Study Group

**Affiliations:** 1 China-Japan Friendship School of Clinical Medicine Capital Medical University Beijing China; 2 National Center for Respiratory Medicine Beijing China; 3 State Key Laboratory of Respiratory Health and Multimorbidity Beijing China; 4 National Clinical Research Center for Respiratory Diseases Beijing China; 5 Institute of Respiratory Medicine Chinese Academy of Medical Sciences Beijing China; 6 Department of Tobacco Control and Prevention of Respiratory Diseases Center of Respiratory Medicine China-Japan Friendship Hospital Beijing China; 7 WHO Collaborating Centre for Tobacco Cessation and Respiratory Diseases Prevention Beijing China; 8 Department of Pulmonary and Critical Care Medicine Shanxi Bethune Hospital Shanxi Academy of Medical Sciences Taiyuan China; 9 Department of Pulmonary and Critical Care Medicine First Affiliated Hospital of Xi’an Jiaotong University Xi’an China; 10 Department of Pulmonary and Critical Care Medicine Tongji Hospital Tongji Medical College, Huazhong University of Science and Technology Wuhan China; 11 Department of Pulmonary and Critical Care Medicine Guizhou Provincial People’s Hospital Guiyang China; 12 Department of Pulmonary and Critical Care Medicine Zhongshan Hospital Fudan University Shanghai China; 13 Department of Pulmonary and Critical Care Medicine First Hospital of China Medical University Shenyang China; 14 State Key Laboratory of Respiratory Disease National Clinical Research Center for Respiratory Diseases, Guangzhou Institute of Respiratory Diseases First Affiliated Hospital, Guangzhou Medical University Guangzhou China; 15 Department of Pulmonary and Critical Care Medicine Second Affiliated Hospital of Zhejiang University School of Medicine Hangzhou China; 16 State Key Laboratory of Biotherapy of China and Department of Respiratory and Critical Care Medicine West China Hospital of Sichuan University Chengdu China; 17 Beijing Key Laboratory of Respiratory and Pulmonary Circulation Disorders Department of Pulmonary and Critical Care Medicine Beijing Chao-Yang Hospital, Capital Medical University Beijing China; 18 Department of Pulmonary and Critical Care Medicine Peking University Third Hospital Beijing China; 19 Department of Respiratory and Critical Care Medicine Beijing Hospital Beijing China; 20 National Center of Gerontology Beijing China; 21 Institute of Basic Medical Sciences Chinese Academy of Medical Sciences School of Basic Medicine, Peking Union Medical College Beijing China; 22 Department of Pulmonary and Critical Care Medicine Center of Respiratory Medicine China-Japan Friendship Hospital Beijing China; 23 Center for Clinical Epidemiology and Evidence-Based Medicine Beijing Children's Hospital Capital Medical University Beijing China; 24 National Center for Children’s Health Beijing China; 25 Department of Stomatology Beijing Chao-Yang Hospital Capital Medical University Beijing China; 26 Department of Epidemiology Beijing Chao-Yang Hospital Capital Medical University Beijing China; 27 Beijing Institute of Respiratory Medicine Beijing China; 28 National Heart and Lung Institute Imperial College London and Royal Brompton and Harefield NHS Trust London United Kingdom; 29 Clinical Trial Service Unit and Epidemiological Studies Unit Nuffield Department of Population Health University of Oxford Oxford United Kingdom; 30 Department of Epidemiology Tulane University School of Public Health and Tropical Medicine New Orleans, LA United States; 31 Data and Project Management Unit Center of Respiratory Medicine China-Japan Friendship Hospital Beijing China; 32 See Acknowledgements

**Keywords:** chronic obstructive pulmonary disease, tobacco dependence, smoking, mediating effects, lung function

## Abstract

**Background:**

Maternal smoking during pregnancy (MSDP) is a known risk factor for offspring developing chronic obstructive pulmonary disease (COPD), but the underlying mechanism remains unclear.

**Objective:**

This study aimed to explore whether the increased COPD risk associated with MSDP could be attributed to tobacco dependence (TD).

**Methods:**

This case-control study used data from the nationwide cross-sectional China Pulmonary Health study, with controls matched for age, sex, and smoking status. TD was defined as smoking within 30 minutes of waking, and the severity of TD was assessed using the Fagerstrom Test for Nicotine Dependence. COPD was diagnosed when the ratio of forced expiratory volume in 1 second to forced vital capacity was <0.7 in a postbronchodilator pulmonary function test according to the 2017 Global Initiative for Chronic Obstructive Lung Disease criteria. Logistic regression was used to examine the correlation between MSDP and COPD, adjusting for age, sex, BMI, educational attainment, place of residence, ethnic background, occupation, childhood passive smoking, residential fine particulate matter, history of childhood pneumonia or bronchitis, average annual household income, and medical history (coronary heart disease, hypertension, and diabetes). Mediation analysis examined TD as a potential mediator in the link between MSDP and COPD risk. The significance of the indirect effect was assessed through 1000 iterations of the “bootstrap” method.

**Results:**

The study included 5943 participants (2991 with COPD and 2952 controls). Mothers of the COPD group had higher pregnancy smoking rates (COPD: n=305, 10.20%; controls: n=211, 7.10%; *P*<.001). TD was more prevalent in the COPD group (COPD: n=582, 40.40%; controls: n=478, 33.90%; *P*<.001). After adjusting for covariates, MSDP had a significant effect on COPD (β=.097; *P*<.001). There was an association between MSDP and TD (β=.074; *P*<.001) as well as between TD and COPD (β=.048; *P*=.007). Mediation analysis of TD in the MSDP-COPD association showed significant direct and indirect effects (direct: β=.094; *P*<.001 and indirect: β=.004; *P*=.03). The indirect effect remains present in the smoking population (direct: β=.120; *P*<.001 and indirect: β=.002; *P*=.03).

**Conclusions:**

This study highlighted the potential association between MSDP and the risk of COPD in offspring, revealing the mediating role of TD in this association. These findings contribute to a deeper understanding of the impact of prenatal tobacco exposure on lung health, laying the groundwork for the development of relevant prevention and treatment strategies.

## Introduction

Chronic obstructive pulmonary disease (COPD) is a global health challenge, ranking among the leading causes of both morbidity and mortality worldwide [1]. It was estimated that COPD affected a substantial portion of the global population with a prevalence rate of 10.3% among individuals, and the number of individuals afflicted with COPD has been on the rise [2]. Notably, China accounts for nearly a quarter of global COPD cases, with a 67% increase in prevalence among individuals aged 40 years and older between 2012 and 2015 [3].

COPD is influenced by various factors, including genetics, lifestyle, environmental factors, and other influencing factors [2-5]. Smoking, recognized as the most significant cause of COPD, leads to its development in approximately half of all smokers [6,7]. The prenatal and perinatal phases are crucial for lung development, with maternal smoking linked to offspring susceptibility to various health issues [8-10]. A systematic review suggested that maternal smoking during pregnancy (MSDP) is associated with an increased risk of COPD [11]. MSDP may directly impact fetal development, influencing the formation of the central nervous system and respiratory system [12]. Given the vulnerability of embryos and fetuses to the external environment, maternal smoking may exert a more pronounced effect on fetal development [13].

Despite widespread recognition of the adverse effects of MSDP on offspring COPD [11], the underlying mechanisms remain incompletely elucidated. Tobacco dependence (TD) is a complex condition influenced by genetic and environmental factors, classified as a mental disorder according to the International Classification of Diseases, and TD should be recognized as a lethal noncommunicable disease [14]. Previous studies have identified TD as an independent risk factor for atherosclerosis [15]. Notably, TD is prevalent among smokers, often associated with heightened smoking intensity and lower cessation rates [16], and approximately 40% of smokers experience impaired lung function and develop COPD [17]. A meta-analysis indicates an elevated risk of smoking and TD in offspring associated with MSDP [18]. Given these associations, our hypothesis posits that TD plays a pivotal mediating role in the association between MSDP and COPD.

Therefore, this study aims to investigate whether TD serves as a mediator in the association between MSDP and the risk of COPD in offspring. To achieve this, we used data from the national cross-sectional China Pulmonary Health (CPH) study [3] and conducted a case-control study to explore the role of TD in the association between MSDP and COPD. The findings will contribute to the development of targeted interventions focused on TD.

## Methods

### Participants and Study Design

The study population was drawn from the national cross-sectional CPH study [3,19,20], the largest study assessing the burden of COPD in China. The CPH study, encompassing a vast nationwide cross-sectional examination, encompassed 57,779 Chinese adults aged 20 years and older across 10 provinces in China. This extensive study incorporated 80 urban and 80 rural areas, using a multistage stratified cluster sampling approach, The details of recruitment for the CPH can be found elsewhere [3]. Trained health workers in local community health centers administered standardized questionnaire surveys to all participants, capturing essential information on sociodemographic status, medical history, lifestyle, and more. The questionnaire information involved in this study can be obtained in Table S1 in Multimedia Appendix 1.

**Figure 1:**
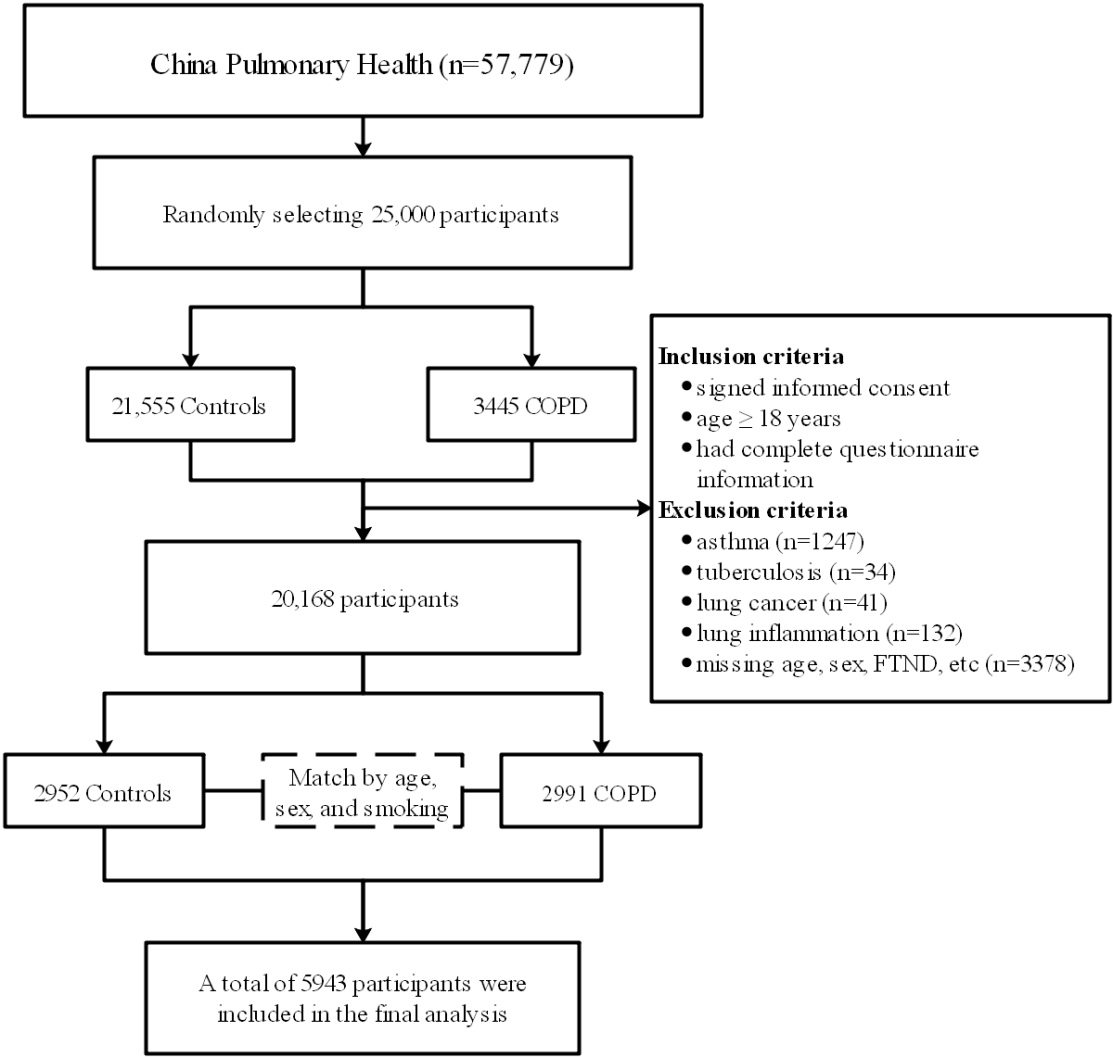
The workflow of study. COPD: chronic obstructive pulmonary disease; FTND: Fagerstrom Test for Nicotine Dependence.

In this study, a case-control design was used, and the workflow is illustrated in Figure 1. Inclusion criteria for the case group were as follows: (1) provision of signed informed consent, (2) age of 18 years or older, and (3) availability of complete questionnaire information. Individuals with other conditions such as asthma, tuberculosis, lung cancer, lung inflammation, and missing information were excluded. Control participants were matched for age, sex, and smoking from cohort. The matching tolerance can be found in Table S2 in Multimedia Appendix 1. Ultimately, a total of 2991 patients in the COPD group and 2952 controls were included in this study.

### Definition of MSDP and Evaluation of TD

MSDP was self-reported through a questionnaire, with all participants asked to respond to the question, “Did your mother smoke during pregnancy? (yes or no).” Upon awakening, smoking within the first 30 minutes was defined as indicative of TD, based on prior study [21]. The severity of TD was assessed using the Fagerstrom Test for Nicotine Dependence (FTND) [22]. FTND is a standardized tool for evaluating the degree of physical nicotine addiction. It consists of six items: (1) How soon after waking do you smoke your first cigarette (within 5 minutes, 6 to 30 minutes, 31 to 60 minutes, and after 60 minutes)? (2) Do you find it difficult to refrain from smoking in places where it is prohibited (yes or no)? (3) Which cigarette would you find most difficult to give up (the first one in the morning or any other)? (4) How many cigarettes do you smoke per day (10 or less, 11 to 20, 21 to 30, and 31 or more)? (5) Do you smoke more frequently during the first hour after waking compared to the rest of the day (yes or no)? and (6) Do you smoke when you are so ill that you are confined to bed most of the day (yes or no)? The FTND generates a total score ranging from 0 to 10, with scores calculated as the sum of individual items. Yes or no items are scored as 0 or 1, and multiple-choice items are scored as 0 to 3. The questionnaire provided a comprehensive assessment of cigarette consumption, compulsion, and dependence. A higher total FTND score indicates a greater degree of TD. All participants reported their smoking status (yes or no). Smoking was defined as having consumed 100 cigarettes in one’s lifetime, encompassing both current and former smokers.

To address potential recall bias in self-reported MSDP, we implemented rigorous quality control measures. Our questionnaire design emphasized clarity and comprehensibility, and interviewers received detailed training to ensure precise definitions of smoking behavior. Memory aids were provided to aid accurate recall, and privacy protection measures were emphasized to minimize bias.

### Definition of COPD

All study participants underwent postbronchodilator pulmonary function tests, including measurements of forced expiratory volume in 1 second (FEV_1_) and forced vital capacity (FVC) and FEV_1_/FVC ratio. COPD was diagnosed in accordance with the criteria set forth by the 2017 Global Initiative for Chronic Obstructive Lung Disease [23] if the participant’s FEV_1_/FVC ratio was <0.7.

### Covariates

This study incorporated several covariates, including age (years), sex (male or female), BMI (calculated as weight in kilograms divided by the square of height in meters), educational attainment (primary and below, junior middle school, senior high school, or bachelor and above), place of residence (urban or rural), ethnic background (Han Chinese or other), occupation (farmer, worker, or other), childhood passive smoking, residential fine particulate matter (PM_2.5_) exposure, history of childhood pneumonia or bronchitis (yes or no), average annual household income, and medical history (coronary heart disease, hypertension, and diabetes).

### Statistical Analysis

For normally distributed continuous variables, descriptive statistics are presented as mean (SD). Categorical variables are expressed as numbers and percentages. The Kolmogorov-Smirnov test was used to assess the normality of continuous variables. Comparisons of continuous variables were conducted using the Mann-Whitney *U* test, while categorical variables were compared using the chi-square test.

Mediation analysis, as outlined by Baron and Kenny [24], was used to investigate the potential mediating role of TD in the association between MSDP and the risk of COPD. The association between MSDP and TD was modeled using logistic regression, whereas the association with FTND scores was assessed using linear regression. The total effect of the initial variable on the outcome was defined as the sum of its direct and indirect effects. The indirect effect was further defined as the product of the initial variable’s impact on the intermediate variable and the intermediate variable’s impact on the outcome, with adjustments made for the initial variable. The statistical significance of the indirect effect was assessed through 1000 repetitions of a bootstrap procedure. Regarding the sensitivity analysis, we conducted a similar analysis by defining TD as a total FTND score ≥4. The sample size for this study was determined using the Monte Carlo method [25]. The details of the sample size calculation process are provided in Table S3 in Multimedia Appendix 1. All statistical analyses were conducted using R (version 4.2.1; R Foundation for Statistical Computing), with the use of packages such as *bruceR*, *interactions*, and *mediation* to estimate mediating effects. A significance level of *P*=.05 (2-tailed) was considered statistically significant.

### Ethical Considerations

The study received approval from the ethics review committee of Beijing Capital Medical University (11-KE-42) and other collaborating institutes. Written informed consent was obtained from all study participants in accordance with the principles outlined in the Declaration of Helsinki. To ensure privacy and confidentiality, the data used in this study underwent anonymization and deidentification processes.

## Results

### Participants

A total of 5943 participants were enrolled in this study, comprising 2991 individuals with COPD and 2952 matched controls. The demographic characteristics of all participants are elaborated in Table 1. In the control group, the mean age was 59.17 (SD 11.44) years, while in the COPD group, it was 59.50 (SD 11.66) years. Among controls, there were 1783 (60.40%) male participants, and in the COPD group, 1816 (60.70%) were male participants. Smoking was observed in 1442 (48.20%) individuals within the COPD group and 1412 (47.80%) individuals in the control group. For matching purposes, no statistically significant differences were detected between the 2 groups in terms of participants’ age, sex, and smoking. However, a higher proportion of mothers in the COPD group reported MSDP compared to the control group (COPD: n=305, 10.20% and controls: n=211, 7.10%; *P*<.001).

**Table 1:** Demographic characteristics of participants in this study.

		Controls (n=2952)		COPD^a^ (n=2991)	*P* value	
Age (years), mean (SD)		59.17 (11.44)		59.50 (11.66)	.27^b^	
**Sex, n (%)**						.82^c^
	Female	1169 (39.60)	1175 (39.30)			
	Male	1783 (60.40)	1816 (60.70)			
**BMI (kg/m^2^), n (%)**						<.001^c^
	<18.5	96 (3.30)	129 (4.30)			
	18.5-25	1682 (57)	1849 (61.80)			
	≥25	1174 (39.80)	1013 (33.90)			
**Education, n (%)**						<.001^c^
	Primary and below	971 (32.90)	1227 (41)			
	Junior middle school	988 (33.50)	987 (33)			
	Senior high school	620 (21)	493 (16.50)			
	Bachelor and above	373 (12.60)	284 (9.50)			
**Place of residence, n (%)**						<.001^c^
	City	1977 (67)	1875 (62.7)			
	Countryside	975 (33)	1116 (37.3)			
**Nation, n (%)**						.02^c^
	Han Chinese	2897 (98.10)	2906 (97.20)			
	Other	55 (1.90)	85 (2.80)			
**Occupation, n (%)**						<.001^c^
	Farmer	794 (26.90)	1172 (39.20)			
	Worker	850 (28.80)	678 (22.70)			
	Other	1308 (44.30)	1141 (38.10)			
**MSDP^d^, n (%)**						<.001^c^
	No	2741 (92.90)	2686 (89.80)			
	Yes	211 (7.10)	305 (10.20)			
**Smoking, n (%)**						.79^c^
	No	1540 (52.20)	1549 (51.80)			
	Yes	1412 (47.80)	1442 (48.20)			
**GOLD^e^, n (%)**						—^f^
	1	—	1685 (56.30)			
	2	—	1103 (36.90)			
	3	—	172 (5.80)			
	4	—	31 (1)			
FEV_1_^g^, mean (SD)		2.67 (0.66)		2.13 (0.70)	<.001^b^	
FEV_1_/FVC^h^ ratio, mean (SD)		80.44 (5.63)		61.18 (9.00)	<.001^b^	
AAHI^i^, mean (SD)		1.34 (2.39)		1.11 (1.18)	<.001^b^	
PM_2.5_^j^, mean (SD)		71.85 (13.64)		73.81 (15.95)	<.001^b^	
**Childhood passive smoking, n (%)**						.049^c^
	No	1184 (40.1)	1276 (42.7)			
	Yes	1768 (59.9)	1715 (57.3)			
**History of childhood pneumonia or bronchitis, n (%)**						<.001^c^
	No	2842 (96.3)	2789 (93.2)			
	Yes	110 (3.7)	202 (6.8)			
**Coronary heart disease, n (%)**						.11^c^
	No	2888 (97.8)	2906 (97.2)			
	Yes	64 (2.2)	85 (2.8)			
**Hypertension, n (%)**						.007^c^
	No	2575 (87.2)	2677 (89.5)			
	Yes	377 (12.8)	314 (10.5)			
**Diabetes, n (%)**						.70^c^
	No	2851 (96.6)	2895 (96.8)			
	Yes	101 (3.4)	96 (3.2)			

^a^COPD: chronic obstructive pulmonary disease.

^b^*P* value for 2-tailed *t* test.

^c^*P* value for chi-square test.

^d^MSDP: maternal smoking during pregnancy.

^e^GOLD: Global Initiative for Chronic Obstructive Lung Disease.

^f^Not available.

^g^FEV_1_: forced expiratory volume in 1 second.

^h^FVC: forced vital capacity.

^i^AAHI (average annual household income) is measured in 10,000 Yuan (a currency exchange rate of 1 Chinese Yuan (CNY)=US $0.16147 is applicable).

^j^PM_2.5_: fine particulate matter.

### Differences of TD in Smokers Between the 2 Groups

As depicted in Table 2, the prevalence of TD was notably higher in the COPD group in comparison to the control group (COPD: n=582, 40.40%; controls: n=478, 33.90%; *P*=.001). Patients with COPD, when contrasted with the control group, exhibited a shorter time interval between waking up and the desire to smoke their first cigarette (Q1: *P*=.004) and encountered greater difficulty refraining from smoking in public places (Q2: *P*=.006). Among the COPD group, 19.40% (n=280) reported a tendency to smoke more frequently during the early hours after waking, whereas in the control group, this behavior was observed in 16.50% (n=233; *P*=.048). Additionally, the proportion of individuals smoking when unwell was higher in the COPD group than in the control group (Q6: *P*<.001). Q3 showed no statistically significant difference between the 2 groups, but with a *P* value <0.1, suggesting the potential presence of marginal effects.

**Table 2:** Differences between the COPD^a^ and control group in the FTND^b^ among smokers.

Questions			Controls (n=1412), n (%)		COPD (n=1442), n (%)		*P* value^c^
**Q1^d^**							.004
	After 60 minutes	784 (55.50)		728 (50.50)			
	31 to 60 minutes	150 (10.60)		132 (9.20)			
	6 to 30 minutes	186 (13.20)		235 (16.30)			
	Within 5 minutes	292 (20.70)		347 (24.10)			
**Q2^e^**							.006
	No	1122 (79.50)		1082 (75)			
	Yes	290 (20.50)		360 (25)			
**Q3^f^**							.06
	Any other	1059 (75)		1035 (71.80)			
	The first one in the morning	353 (25)		407 (28.20)			
**Q4^g^**							.62
	10 or less	1228 (87)		1247 (86.50)			
	11 to 20	141 (10)		139 (9.60)			
	21 to 30	19 (1.30)		22 (1.50)			
	31 or more	24 (1.70)		34 (2.40)			
**Q5^h^**							.048
	No	1179 (83.50)		1162 (80.60)			
	Yes	233 (16.50)		280 (19.40)			
**Q6^i^**							<.001
	No	1221 (86.50)		1168 (81)			
	Yes	191 (13.50)		274 (19)			
Total score			1.92 (2.03)		2.25 (2.14)		<.001
**TD^j^**							<.001
	No	934 (66.10)		860 (59.60)			
	Yes	478 (33.90)		582 (40.40)			

^a^COPD: chronic obstructive pulmonary disease.

^b^FTND: Fagerstrom Test for Nicotine Dependence.

^c^*P* value for chi-square test.

^d^Q1: How soon after you wake up do you smoke your first cigarette?

^e^Q2: Do you find it difficult to refrain from smoking in places where it is forbidden?

^f^Q3: Which cigarette would you find most difficult to give up?

^g^Q4: How many cigarettes do you smoke per day?

^h^Q5: Do you smoke more frequently during the first hour after waking compared to the rest of the day?

^i^Q6: Do you smoke when you are so ill that you are confined to bed most of the day?

^j^TD: tobacco dependence.

### Direct and Indirect Effects of MSDP on COPD in All Participants

The results of the mediation analysis for all participants are presented in Table 3. After adjusting for age, sex, BMI, educational attainment, place of residence, ethnicity, occupation, childhood passive smoking, residential PM_2.5_ exposure, history of childhood pneumonia or bronchitis, average annual household income, and medical history (coronary heart disease, hypertension, and diabetes), the total effect of MSDP on COPD was statistically significant (β=.097; *P*<.001). Furthermore, the direct effect of MSDP on COPD remained significant after adjusting for TD and other covariates (β=.094; *P*<.001). The indirect effect of TD was also found to be statistically significant (β=.004; *P*=.03). The sensitivity analysis revealed similar results (Table S4 in Multimedia Appendix 1).

**Table 3:** Mediating effect of TD^a^ (mediating variable) on the association between MSDP^b^ (independent variable) and COPD^c^ (dependent variable) in all participants^d^.

Path	β (SE)	*P* value
MSDP on COPD^e^	.097 (0.023)	<.001
MSDP on TD^f^	.074 (0.017)	<.001
TD on COPD given MSDP^g^	.048 (0.018)	.007
Indirect: MSDP on COPD^h^	.004 (0.002)	.03
Direct: MSDP on COPD given TD^i^	.094 (0.023)	<.001

^a^TD: tobacco dependence.

^b^MSDP: maternal smoking during pregnancy.

^c^COPD: chronic obstructive pulmonary disease.

^d^All analyses adjusted for age, sex, BMI, educational attainment, place of residence, ethnicity, occupation, childhood passive smoking, residential PM_2.5_ exposure, history of childhood pneumonia or bronchitis, average annual household income, and medical history (coronary heart disease, hypertension, and diabetes).

^e^Total effect of independent variables on dependent variables.

^f^Coefficients of independent variables on mediating variables after adjustment for covariates.

^g^Coefficients of mediating variables on dependent variables after adjusted for covariates and IVs.

^h^Indirect effect of independent variables on dependent variables.

^i^Direct effect of independent variables on dependent variables.

### Direct and Indirect Effects of MSDP on COPD in Smokers

The results of the mediation analysis for smokers are presented in Table 4. In this segment, all analyses were adjusted for age, sex, BMI, educational attainment, place of residence, ethnicity, occupation, childhood passive smoking, residential PM_2.5_ exposure, history of childhood pneumonia or bronchitis, average annual household income, and medical history (coronary heart disease, hypertension, and diabetes). Among smokers, both the total effect and direct effect (β) of MSDP on COPD were .123 and .120, respectively. Additionally, the indirect effect of TD was found to be statistically significant (β=.003; *P*=.03). The sensitivity analysis revealed similar results (Table S5 in Multimedia Appendix 1).

**Table 4:** Mediating effect of TD^a^ (mediating variable) on the association between MSDP^b^ (independent variable) and COPD^c^ (dependent variable) in smokers^d^.

Path	β (SE)	*P* value
MSDP on COPD^e^	.123 (0.032)	<.001
MSDP on TD^f^	.050 (0.031)	<.001
TD on COPD given MSDP^g^	.059 (0.019)	.004
Indirect: MSDP on COPD^h^	.003 (0.002)	.03
Direct: MSDP on COPD given TD^i^	.120 (0.031)	<.001

^a^TD: tobacco dependence.

^b^MSDP: maternal smoking during pregnancy.

^c^COPD: chronic obstructive pulmonary disease.

^d^All analyses adjusted for age, sex, BMI, educational attainment, place of residence, ethnicity, occupation, childhood passive smoking, residential PM_2.5_ exposure, history of childhood pneumonia or bronchitis, average annual household income, and medical history (coronary heart disease, hypertension, and diabetes).

^e^Coefficients of independent variables on dependent variables after correction for covariates.

^f^Coefficients of independent variables on mediating variables after adjustment for covariates.

^g^Coefficients of mediating variables on dependent variables after correction for covariates and IVs.

^h^Indirect effect of independent variables on dependent variables.

^i^Direct effect of independent variables on dependent variables.

## Discussion

### Principal Findings

The aim of this study was to investigate whether TD plays a role in the association between MSDP and COPD in offspring. A case-control study was conducted within the Chinese population. It was the first study to observe that MSDP has both a direct impact on COPD and an indirect influence mediated through TD. This mediation effect persists among the smoking population, and sensitivity analysis confirms the stability of these results.

It is well-established that smoking represents the primary risk factor for COPD, and an increasing body of evidence suggests that early life tobacco exposure plays a role in the onset and progression of COPD [26]. MSDP, as highlighted by Jaakkola et al [27], exerts substantial adverse effects on conditions such as asthma, chronic bronchitis, and chronic respiratory symptoms. Nevertheless, limited research has delved into the impact of prenatal tobacco smoke exposure on the development of COPD in later adulthood. This study, however, reveals an association between MSDP and the risk of COPD in offspring. Early life tobacco exposure has enduring consequences on lung function in offspring, with suboptimal intrauterine conditions leading to disturbances in lung development. As a result, affected individuals exhibit diminished lung function at birth, which often persists throughout their lifetime. This, in turn, elevates the risk of subsequent COPD, particularly in childhood wheeze disorders and genetically susceptible individuals [28]. To curb the current upward trend in COPD incidence, it is imperative to mitigate lung development risks by enhancing antenatal and neonatal care and reducing exposure to environmental pollutants, including passive tobacco smoke, both prenatally and postnatally.

In this study, MSDP emerges as a risk factor for TD in offspring, a finding consistent with prior research. Previous studies have established that individuals with a history of MSDP face an elevated risk of TD in adolescence and adulthood [18]. The precise mechanism underlying the connection between MSDP and offspring TD remains elusive. One plausible mechanism could involve the neurotoxic effects of harmful compounds present in tobacco smoke, which can readily traverse the placenta, potentially leading to smoking behavior and dependence in offspring [29]. Another conceivable mechanism is that MSDP is linked to preterm birth [30] and low birth weight [31], both of which could predispose offspring to various health challenges. Furthermore, it is essential for health care professionals to recognize that TD may be linked to early life tobacco exposure. Therefore, children exposed to MSDP should receive comprehensive education on topics such as diet, exercise, and smoking avoidance to mitigate their risk of developing COPD in adulthood.

This study revealed a direct association between TD and COPD. Previous research has identified smoking as one of the primary risk factors for COPD, causing inflammation and damage to the airways and lung tissue, ultimately leading to the development of COPD [32]. Recent studies have further suggested the correlation between TD and COPD [33]. Additionally, other studies suggest that TD may exacerbate the severity and progression of COPD [34]. Among individuals diagnosed with COPD, the presence of TD makes quitting smoking more challenging [35]. Thus, this study underscores the critical importance of addressing TD in the management and mitigation of COPD.

To our knowledge, we have discovered for the first time in this study that TD plays a mediating role in the association between MSDP and COPD. However, the specific reasons underlying this mediating mechanism remain unclear. There are several possible mechanisms to consider. First, genetic factors may be implicated, as MSDP has the potential to induce persistent epigenetic changes until adolescence [36]. These genetic alterations may contribute to the development of TD [37]. Consequently, TD can exacerbate challenges in smoking cessation and the progression of COPD [38]. Second, environmental factors may also be involved. Mothers who smoke during pregnancy may continue smoking during infancy, creating an early growth environment that promotes the risk of TD in offspring during lung development [39,40], subsequently leading to COPD. Our results highlight that TD serves as a partial mediator in the connection between MSDP and offspring COPD. Consequently, it becomes imperative to consider TD when addressing MSDP and smoking cessation efforts in offspring. Prior studies have advocated smoking cessation strategies for TD smokers, incorporating the use of medications such as varenicline, nicotine replacement therapy products, and bupropion [41]. Hence, clinicians should be cognizant of the necessity for appropriate smoking cessation interventions among individuals displaying signs of TD, ultimately contributing to the reduction of COPD risk.

### Limitations

This study bears significant implications as it substantiates the partial mediating role of TD in the link between MSDP and COPD, underlining the importance of developing interventions targeting TD to mitigate the incidence of COPD in offspring. Nonetheless, it is essential to acknowledge the study’s limitations. First, the data concerning MSDP relied on questionnaire responses, introducing a potential source of recall bias. Due to the presence of recall bias, we cannot entirely rule out the possibility of uncertainty or erroneous memory in participants when reporting maternal smoking behavior during pregnancy. To enhance the accuracy of capturing MSDP information, future studies may consider using prospective cohort designs. Second, the absence of genetic data represents a significant limitation in this study. Despite the inclusion of various participant covariates, the intricate role of genetic factors in influencing COPD was not considered due to data constraints. Future research endeavors could overcome this limitation by integrating comprehensive genetic analyses into the study design. Finally, our classification of nonsmokers as nonnicotine-dependent during the overall analysis might introduce some bias. To fortify the robustness of our findings, we conducted a parallel subgroup analysis among the smoking population that consistently demonstrated the robustness of our results.

### Conclusions

MSDP exerts an adverse influence on the risk of COPD in offspring, and TD plays a partially mediating role in this association. These findings underscore the potential for clinicians to mitigate the impact of MSDP on COPD in offspring by addressing TD. Consequently, early intervention strategies aimed at reducing TD become imperative in the endeavor to mitigate the risk of COPD.

## Appendix

Multimedia Appendix 1 Supplementary tables.

## Abbreviations

COPD: chronic obstructive pulmonary disease
CPH: China Pulmonary Health
FEV1: forced expiratory volume in 1 second
FTND: Fagerstrom Test for Nicotine Dependence
FVC: forced vital capacity
MSDP: maternal smoking during pregnancy
PM2.5: fine particulate matter
TD: tobacco dependence

## Data Availability

The data sets generated and analyzed during this study are available from the corresponding author on reasonable request.
