# Supplementary material for: Mediating Effect of Tobacco Dependence on the Association Between Maternal Smoking During Pregnancy and Chronic Obstructive Pulmonary Disease: Case-Control Study
Source: JMIR Public Health Surveill. 2024 Feb 22;10:e53170. doi: 10.2196/53170 (PMC10921321; doi:10.2196/53170)
Supplement: Multimedia Appendix 1 [file publichealth_v10i1e53170_app1.docx]

**Table S1.** Questionnaire information relevant to the study.

| var | Questions |
| --- | --- |
| Q1 | Were you exposed to passive smoking during childhood? |
| Q2 | Do you have a history of childhood pneumonia or bronchitis? |
| Q3 | What is the total ana income of your household? |
| Q4 | How many individuals are there in your household? |
| Q5 | Have you ever been diagnosed with coronary heart disease by doctor? |
| Q6 | Have you been diagnosed with hypertension by doctor? |
| Q7 | Have you been diagnosed with diabetes by doctor? |
| Q8 | What is your age? |
| Q9 | What is your sex? |
| Q10 | What is your height (cm)? |
| Q11 | What is your weight (Kg)? |
| Q12 | What is your level of education: 1=Primary school or below 2=Junior high school 3=High school/Technical school 4=College/University 5=Graduate school |
| Q13 | Did your mother smoke during pregnancy? (Yes/No) |
| Q14 | Do you smoke? (Yes/No) |
| Q15 | Have you been diagnosed with COPD by doctor? |
| Q16 | How soon after waking up do you typically smoke your first cigarette? 1=Within 5 minutes 2=6 to 30 minutes 3=31 to 60 minutes 4=After 60 minutes |
| Q17 | Do you find it difficult to refrain from smoking in places where it is forbidden? (Yes/No) |
| Q18 | Which cigarette would you hate most to give up? 1=The first one in the morning 2=Any other |
| Q19 | Do you smoke more during the first hour after waking up compared to other times? (Yes/No) |
| Q20 | Do you smoke when you are bedridden due to illness? (Yes/No) |
| Q21 | On average, how many cigarettes do you smoke per day during your smoking years? (Number of cigarettes per day) |
| Q22 | Post-bronchodilator FVC (Forced Vital Capacity) |
| Q23 | Post-bronchodilator FEV1 (Forced Expiratory Volume in 1 second) |
| Q24 | Post-bronchodilator FEV1/FVC ratio |
| Q25 | Ethnicity: 1=Han Chinese 2=non-Han |
| Q26 | What is your occupation? |

**Table S2.** Matching tolerance for matched variables.

| Var | Match Tolerance |
| --- | --- |
| Age (years) | 2 |
| Sex (male/female) | 0 |
| Smoking (yes/no) | 0 |

**Table S3.** Sample size calculation process.

| Software | https://schoemanna.shinyapps.io/mc_power_med/ |
| --- | --- |
| a | 0.120 |
| b | 0.056 |
| c | 0.097 |

With 1000 repetitions of the calculation, statistical power reached 0.82 when the sample size was set at 2500 individuals. TD: Tobacco dependence; MSDP: maternal smoking during pregnancy; FTND: Fagerstrom Test for Nicotine Dependence. a, b, and c were generated based on pre-survey data.

c: MSDP on COPD

a: MSDP on TD

b: TD on COPD given MSDP.

**Table S4.** Mediating effect of tobacco dependence on the relationship between MSDP and COPD in all participants.

| Model | Path | β | SE | P |
| --- | --- | --- | --- | --- |
| IV: MSDP  MV: TD  DV: COPD | MSDP on COPD ^(c)^ | 0.097 | 0.023 | < .001 |
|  | MSDP on TD ^(a)^ | 0.079 | 0.015 | < .001 |
|  | TD on COPD given MSDP ^(b)^ | 0.046 | 0.020 | .027 |
|  | Indirect: MSDP on COPD ^(ab)^ | 0.004 | 0.002 | .035 |
|  | Direct: MSDP on COPD given TD ^(c’)^ | 0.094 | 0.023 | <.001 |

β: coefficients; SE: standard error; IV: independent variable; MV: mediating variable; DV: dependent variable; COPD: Chronic obstructive pulmonary disease; TD: Tobacco dependence; MSDP: maternal smoking during pregnancy; FTND: Fagerstrom Test for Nicotine Dependence; ^a^: Coefficients of independent variables on mediator variables after adjustment for covariates; ^b^: Coefficients of mediating variables on dependent variables after adjusted for covariates and independent variables; ^c^: Total effect of independent variables on dependent variables; ^c’^: Direct effect of independent variables on dependent variables; ^ab^: Indirect effect of independent variables on dependent variables. All analysis adjusted for age, sex, BMI, educational attainment, place of residence, ethnicity, occupation, childhood passive smoking, residential PM2.5 exposure, history of childhood pneumonia or bronchitis, average annual household income, and medical history (coronary heart disease, hypertension, diabetes).

**Table S5.** Mediating effect of tobacco dependence on the relationship between MSDP and COPD in smokers.

| Models | Path | β | SE | P |
| --- | --- | --- | --- | --- |
| IV: MSDP  MV: TD  DV: COPD | MSDP on COPD ^(c)^ | 0.123 | 0.023 | < .001 |
|  | MSDP on TD ^(a)^ | 0.082 | 0.028 | < .001 |
|  | TD on COPD given MSDP ^(b)^ | 0.055 | 0.021 | .002 |
|  | Indirect: MSDP on COPD ^(ab)^ | 0.005 | 0.002 | .016 |
|  | Direct: MSDP on COPD given TD ^(c’)^ | 0.119 | 0.031 | <.001 |

β: coefficients; SE: standard error; IV: independent variable; MV: mediating variable; DV: dependent variable; COPD: Chronic obstructive pulmonary disease; TD: Tobacco dependence; MSDP: maternal smoking during pregnancy; FTND: Fagerstrom Test for Nicotine Dependence; ^a^: Coefficients of independent variables on mediator variables after adjustment for covariates; ^b^: Coefficients of mediating variables on dependent variables after adjusted for covariates and independent variables; ^c^: Total effect of independent variables on dependent variables; ^c’^: Direct effect of independent variables on dependent variables; ^ab^: Indirect effect of independent variables on dependent variables. All analysis adjusted for age, sex, BMI, educational attainment, place of residence, ethnicity, occupation, childhood passive smoking, residential PM2.5 exposure, history of childhood pneumonia or bronchitis, average annual household income, and medical history (coronary heart disease, hypertension, diabetes).
